# Calcium Ion Mixing Modes Govern Membrane Fouling Mitigation During Membrane-Based Recovery of Extracellular Polymeric Substances

**DOI:** 10.3390/membranes15060169

**Published:** 2025-06-05

**Authors:** Da-Qi Cao, Yi-Xuan Song, Yun-Feng Wu, Guri Yihuo, Jing-Yi Jin

**Affiliations:** 1 Sino-Dutch R&D Centre for Future Wastewater Treatment Technologies, Beijing University of Civil Engineering and Architecture, Beijing 100044, China; 2 Key Laboratory of Urban Stormwater System and Water Environment, Beijing University of Civil Engineering and Architecture, Beijing 100044, China

**Keywords:** extracellular polymeric substance, membrane fouling, mixing mode, membrane fouling mitigation, calcium ion

## Abstract

Recycling extracellular polymeric substances (EPSs) from excess sludge in wastewater treatment plants has garnered significant research attention. Membrane separation offers a promising approach for EPS concentration; however, membrane fouling remains a critical challenge. Previous studies demonstrate that Ca^2+^ addition effectively mitigates membrane fouling. This study reveals that Ca^2+^ mixing modes govern membrane fouling in the dead-end ultrafiltration of both the practical EPS and model EPS [sodium algiante (SA)]. The interaction mechanisms between Ca^2^^+^ and the EPS under varied mixing conditions and their impact on filtration performance were systematically investigated. At a low Ca^2+^ concentration, the addition sequence critically influenced colloidal particle sizes formed via Ca^2^^+^-EPS interactions, altering the cake layer structure governing filtration resistance; these effects diminished at higher Ca^2+^ concentrations. In suspensions prepared by adding EPS to Ca^2+^ solution (EPS-Ca), a portion of the EPS became encapsulated within an EPS-Ca layer formed through Ca^2+^ EPS binding, reducing free EPS concentration and enlarging colloidal aggregates. This encapsulation reduced EPS-mediated membrane fouling, thereby lowering filtration resistance. Conversely, in suspensions prepared by adding Ca^2+^ to EPS solution (Ca-EPS), more complete Ca^2+^ EPS interactions formed a dense crosslinked structure with smaller colloids on membrane surfaces, intensifying fouling and resistance. Additionally, EPS-Ca exhibited higher compressibility than Ca-EPS, though both exhibited comparable filtration resistance under high-pressure conditions. These results offer critical insights into optimizing EPS ultrafiltration concentration to mitigate membrane fouling through Ca^2+^ addition strategies.

## 1. Introduction

The recovery of extracellular polymeric substances (EPSs) from excess sludge in wastewater treatment has become an important research direction [1,2,3,4,5,6,7]. EPSs facilitate the attachment and colonization of microorganisms onto membranes or sludge in activated sludge and biofilm wastewater treatment methods, enhancing pollutant removal and improving the settling of suspended solids by promoting particle aggregation [8,9,10]. EPSs can capture and adsorb pollutants such as heavy metals [2,5], organic compounds [11], and pharmaceuticals [12,13]. EPSs bind and retain nutrients such as nitrogen and phosphorus in wastewater, regulating them to release into the water [8,14,15,16]. Furthermore, EPSs demonstrates the potential for sustainable development through a wide range of applications, including eco-friendly building materials [17], microbial fuel cells [8,18], biosensors [19], ecological construction [20,21] and drug delivery systems in biomedicine [22]. Therefore, the recycling and utilization of EPSs are of great significance to sustainable development.

However, low-energy and efficient thickening technologies, which is a bottleneck for sustainable EPS recovery, are urgently needed for EPS solution with low concentration recovered from excess sludge [4,5,23,24,25]. Traditional concentration methods, such as freezing [26] and vacuum-drying [27], have high energy consumption, making EPS recovery economically unattractive [2,4,5,23,24,25]. Membrane separation technology has gained attention because of the non-alteration of the biological activity of EPSs and the absence of secondary contamination, including, for example, pharmaceutical polysaccharides and biologically active ingredients from Cortex Eucommiae [28], yolk immunoglobulins for animal-derived food preparation [29], and the removal of organic pollutants [30]. However, membrane fouling leads to extremely high filtration resistance, which makes it difficult to further reduce the water content in EPS solution [2,4,5,6,23,24]. Therefore, decreasing membrane fouling in the membrane separation process is the key to realizing the low-energy and high-efficiency recovery of EPS.

Researchers have proposed a variety of methods to mitigate membrane fouling, for example, coagulation/flocculation [31] and ozone oxidation [32] as pretreatment processes and membrane surface modification [33]. Recently, studies have shown that multivalent metal ions (MMIs) can significantly reduce the filtration resistance of biological macromolecules such as practical EPSs and model EPSs (sodium alginate (SA)) [3,4,5,6,23,24,34,35,36,37]. Due to their strong coordination and bridging effects, MMIs bind with EPSs, thereby altering their deposition behavior and cake structure on the membrane surface to mitigate membrane fouling [38,39,40,41]. The substance concentration and the type of added metal ions significantly affect membrane fouling [42,43,44,45]. Table S1 summarizes the filtration characteristics of sodium alginate (SA) with Ca^2+^ addition across different membrane filtration processes, revealing that filtration resistance depends on multiple variables including membrane type, applied pressure, pH, SA molecular weight/concentration, and Ca^2+^ concentration. Specifically, the initial rate of membrane fouling demonstrates a biphasic response to Ca^2+^ concentration, exhibiting an increase with elevated Ca^2^^+^ levels until reaching a critical threshold, beyond which the fouling rate progressively declines [39,40,46,47,48]. Paradoxically, substantial evidence indicates that Ca^2+^ supplementation may conversely exacerbate membrane fouling through ionic bridging mechanisms. Specifically, calcium dosing has been shown to enhance the aggregation propensity of extracellular polymeric substances (EPSs), particularly model compounds like sodium alginate (SA). This ionic interaction fundamentally alters membrane filtration dynamics by accelerating flux decline, reducing fouling reversibility, and modifying solute rejection characteristics [49,50,51].

Given this theoretical gap, we discuss that the interfacial interaction dynamics between EPS and Ca^2+^ solutions—particularly their mixing protocol—may fundamentally shape hydraulic resistance evolution during membrane filtration. This critical parameter remains conspicuously underexplored in current fouling mitigation research despite its potential to dictate colloidal aggregation pathways. Therefore, the systematic investigation of this phenomenon not only advances our theoretical understanding for a deeper understanding of the membrane fouling mitigation mechanism but also offers practical guidance for the concentration process of EPSs.

This study systematically examined the ultrafiltration (UF) performance of EPS solutions under varied mixing modes with Ca^2+^ solutions, evaluating factors including the mixing sequence with Ca^2+^, concentration, operational pressure, dosing volume, and flow rate. Complementary investigations using model EPSs (sodium alginate, SA) were further carried out. Finally, the structure formation mechanisms of EPS-Ca^2+^ interaction modes were analyzed using Fourier-transform infrared spectroscopy (FTIR) spectra, transmission electron microscopy (TEM) images and selected area electron diffraction patterns, and scanning electron microscope (SEM) elemental analysis.

## 2. Materials and Methods

### 2.1. Materials

SA (MW = 12–80 kDa) and CaCl_2_·6H_2_O were all purchased from Sigma-Aldrich (St. Louis, MO, USA). UF membrane (MWCO = 10 kDa) was sourced from Millipore Corp., Billerica, MA, USA. Ultrapure deionized (DI) water (resistivity ≥ 18.2 MΩ) was obtained by purifying tap water using a laboratory Arium Comfort II ultrapure water system (Sartorius Corp., Göttingen, Germany). The cation exchange resin (CER, Amberlite IR 120 Na) was obtained from Rohm and Haas Corp., Philadelphia, PA, USA. The excess sludge was collected from a self-constructed sequencing batch reactor (SBR) wastewater treatment process. After settling at room temperature for 24 h, the sludge was stored in a refrigerator at 4 °C. The measured proportion of volatile solids (VSs) to total solids (TSs) (i.e., VS/TS) in the sludge was 72.3 ± 0.36%.

### 2.2. EPS Extraction

The practical EPS was extracted from the excess sludge using the CER method [23]. Initially, 37.5 mL of sludge (suspended solid of 1 g) was centrifuged at 4000× *g* for 20 min, and the supernatant was discarded to remove impurities. The sludge was then resuspended in deionized water to a final volume of 200 mL. Next, the corresponding CER (70 g/g VS) was added, and the mixture was stirred for 4 h. The mixture was then divided into 50 mL centrifuge tubes and centrifuged again at 4000× *g* for 20 min. The supernatant was collected and placed in a 3500 Da dialysis bag, where it was dialyzed against deionized water for 24 h at a volume ratio of 1:9. This process was repeated twice to remove metal ions and other impurities. Finally, EPS powder was obtained by freeze-drying. This method ensures a sufficient amount of EPS powder for the experiments, guarantees batch consistency, and maintains the stability of EPS characteristics across all membrane filtration experiments.

### 2.3. Sample Preparation

EPS solutions [including the natural EPS and model EPS (SA)] were prepared in ultrapure water by stirring for 12 h at 25 °C. CaCl_2_ solutions were obtained by dissolving metal chlorides in ultrapure water with 3 h stirring. The suspended substance formed by adding CaCl_2_ solution to EPS solution (Ca^2+^ → EPS) with stirring for 6 h was named Ca-EPS. The suspended substance formed by adding EPS solution to CaCl_2_ solution (EPS → Ca^2+^) with stirring for 6 h was named EPS-Ca. Table 1 presents detailed experimental parameters and characteristics of the sample preparation.

To investigate the addition order between metal ions and the EPS (maintaining the final concentration at 1.0 g/L EPS and 1 mM/5 mM Ca^2+^), 50 mL of CaCl_2_ solution (5 mM/25 mM) was injected into 200 mL EPS solution (1.25 g/L) at a 25 mL/min flow rate. On the other hand, 200 mL EPS solution (1.25 g/L) was injected into 50 mL of CaCl_2_ solution (5 mM/25 mM) at a 25 mL/min flow rate. Pressure-dependent addition sequence experiments (maintaining the final concentration at 1.0 g/L EPS and 1 mM Ca^2+^) were carried out under varying pressures (20–100 kPa).

For evaluating concentration–volume effects (maintaining the final concentration at 1.0 g/L EPS and 5 mM Ca^2+^), three CaCl_2_ solutions (0.5 M/10 mL, 1 M/5 mL, and 1.75 M/2.86 mL) were added via a peristaltic pump (25 mL/min) to 1000 mL EPS solution (1.0 g/L). Flow rate impacts were tested by injecting 10 mL CaCl_2_ solution (0.5 M) into 1000 mL EPS solution (1.0 g/L) at 5, 15, and 25 mL/min using a constant-flow pump with continuous stirring.

All mixtures underwent 6 h stirring (300 rpm, 25 °C) until reaction completion, followed by pH measurement.

### 2.4. Filtration Apparatus

**Figure 1:**
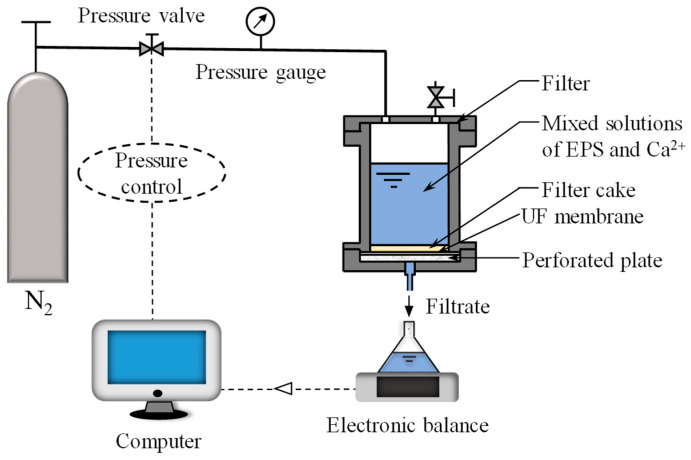
Schematic diagram of dead-end filtration apparatus.

Dead-end filtration experiments were performed under constant pressure (20–100 kPa) using a filter (Model 8400, Millipore) with an effective area of 19.6 cm^2^. Figure 1 shows a schematic diagram of the filtration apparatus. A constant pressure was applied using N_2_ gas and was controlled using an automatic pressure-regulating valve. The filtrate was collected in a reservoir placed on an electronic balance connected to a personal computer for recording mass versus time data. The weights were converted to volumes using density correlation. The filtration rate was obtained by the numerical differentiation of the volume versus time data.

### 2.5. Evaluation of Filtration Behaviors

The behaviors of flux declining were analyzed by the Ruth cake filtration model [3,23,24]. Theoretically, the relationship between the reciprocal of the filtration rate (d*t*/d*v*) and the cumulative filtrate volume collected per unit effective membrane area, *v*, is linear by the Ruth filtration equation [3,5].

(1) $$\frac{dt}{dv}-{\left(\frac{dt}{dv}\right)}_{m}=\frac{2}{K_{v}}v$$

Here, *t* is the filtration time, and *v* is equivalent to the cumulative filtrate volume collected per unit effective membrane area. (d*t*/d*v*)_m_ represents the membrane flow resistance and is an intercept on the (d*t*/d*v*) axis according to the Ruth plots of (d*t*/d*v*) as a function of *v*, which is the value of the reciprocal of the filtration rate at the start of filtration without formed cake. *K*_v_ is the Ruth filtration coefficient, indicating the filterability of the feed as defined by

(2) $$K_{v}=\frac{2p\left(1-ms\right)}{\mu \rho \alpha_{av}}$$

Here, *p* represents the applied filtration pressure, *m* is the ratio of the mass of the wet cake to the mass of particles, colloids, or polymers in the cake, and *s* is the mass fraction of the particles, colloids, or polymers contained in suspension, *μ* is the filtrate viscosity, *ρ* is the filtrate density, and *α*_av_ is the cake average specific resistance. As the term (1 − *ms*) may be approximated by unity in filtrations conducted with dilute particles, colloids, or polymers, Equation (2) reduces to

(3) $$K_{v}=\frac{2p}{\mu \rho \alpha_{av}}$$

According to the power law relationship [52], the relationship between *α*_av_ and *p* is approximately linear, as follows:

(4) $$\alpha_{av}=\alpha p^{n}$$

where α and *n* are empirical constants, and *n* is referred to as the compressibility coefficient.

### 2.6. Analytical Methods

#### 2.6.1. Determination of Calcium Ion

In total, 30 mL of the thoroughly mixed aqueous solution was transferred to a graduated cylinder and allowed to settle undisturbed for 24 h. A 5 mL aliquot of the supernatant was acidified with nitric acid and then filtered through 0.45 μm membrane. The free Ca^2^^+^ concentration in the EPS-Ca^2^^+^ mixture was quantified using an inductively coupled plasma (ICP) spectrometer (ICAP 7000 Series, Thermo Scientific, Waltham, MA, USA) to determine the amount of Ca^2^^+^ bound to EPS.

#### 2.6.2. Dynamic Light Scattering Analysis

The mixed aqueous solution of the EPS and metal ions was stirred for over 12 h to ensure complete interaction. All samples were transferred to standard cuvettes filled to the calibration mark. The size distributions of colloidal/polymeric particles formed by the interaction between the EPS and Ca^2+^ were measured using a Nano ZS 90 Zetasizer dynamic light scattering (DLS) analyzer (Malvern, UK).

#### 2.6.3. FTIR Spectroscopy Analysis

Freeze-dried powders of particles or colloids were prepared with an FD-1A-50 vacuum lyophilizer (Beijing Boyikang Laboratory Instruments, Beijing, China). Samples were homogenized with KBr at a 1:100 mass ratio and oven-dried overnight at 120 °C. The dried mixtures were ground in an agate mortar, compressed into transparent pellets, and analyzed using Nicolet iS5 FTIR spectroscopy (Nicolet is5, Thermo Scientific, Waltham, MA, USA) to assess functional group changes in the EPS before and after Ca^2^^+^ interaction.

#### 2.6.4. SEM Analysis

To evaluate the structure of the cake formed on the membrane, the surface and cross-section images of filter cake were obtained using SEM (Zeiss G300, Oberkochen, Germany) at 5 kV or 15 kV after the pretreatment of gold spraying for 10 min under vacuum. Prior to imaging, samples were dried in a DNP-9272 electric thermostatic oven (Shanghai Jinghong, Shanghai, China) at 35 °C.

To analyze the surface elemental distribution of the cake, the samples were dried for 48 h using vacuum freeze-drying (FD-1A-50, Boyikang, Beijing, China), followed by cryo-drying in liquid nitrogen to prepare their cross-sections. Following gold sputter-coating (10 min), the elemental changes in the EPS before and after Ca^2^^+^ interaction were analyzed using low-vacuum SEM (200–7000 V) coupled with an Oxford Xplore30 energy-dispersive X-ray spectrometer (EDS) system. Five random regions per sample were imaged and analyzed.

#### 2.6.5. TEM Analysis

Liquid samples (sonicated for 5 min at 25 °C to ensure dispersion homogeneity) were drop-cast onto carbon-coated TEM grids. Grids were air-dried to evaporate residual solvents before analysis. Changes in the microstructure of the EPS before and after Ca^2^^+^ interaction was characterized using high-resolution TEM with a JEM-F200 instrument (JEOL, Tokyo, Japan) operated at 200 kV. Multi-scale imaging (10 nm to 5 nm resolutions) and selected area electron diffraction (SAED) at 5 nm were conducted to capture hierarchical features spanning gel networks to nanoparticles and nanostructures.

## 3. Results and Discussion

### 3.1. Filtration Behaviors of EPS Solutions for Various Mixing Modes of Ca^2+^

**Figure 2:**
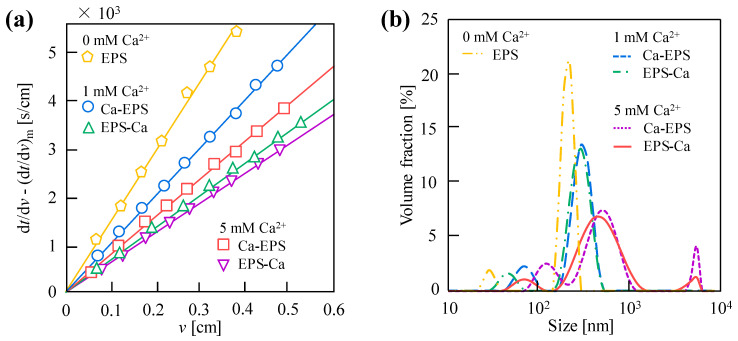
Effect of Ca^2^^+^ addition order (0, 1, 5 mM) on (**a**) ultrafiltration (UF) behavior and (**b**) particle size distribution of extracellular polymeric substance (EPS), Ca-EPS, and EPS-Ca. Here, filtration of 1.0 g/L EPS concentration was conducted at 20 kPa using a 10 kDa UF membrane; EPS-Ca, suspended substance formed by adding EPS solution to Ca^2+^ solution (EPS → Ca^2+^); Ca-EPS, suspended substance formed by adding Ca^2+^ solution to EPS solution (Ca^2+^ → EPS).

Figure 2a compares UF behaviors of the EPS, EPS-Ca, and Ca-EPS under different mixing orders. For the additions of 1 mM and 5 mM Ca^2+^, the filtration resistance of EPS-Ca was significantly lower than Ca-EPS. All Ca^2+^-added systems exhibited reduced filtration resistance compared to Ca^2+^-free controls, with resistance inversely proportional to Ca^2+^ concentration regardless of the addition order and concentration of Ca^2+^. The filtration resistance (membrane fouling) decreased with increasing Ca^2+^ concentration across the studied range, independent of the Ca^2+^ addition order, attributed to EPS-Ca^2+^ interactions forming large aggregates. For detailed Ca^2+^ concentration effects, refer to our prior studies [3,23]. Figure 2b shows particle/colloid size distributions for the EPS, EPS-Ca, and Ca-EPS. The size of EPS-Ca and Ca-EPS was larger than that of the EPS, consistent with reduced filtration resistance. All Ca^2+^ containing systems displayed right-shifted particle size peaks, regardless of the addition order or concentration, confirming Ca^2+^-induced charge neutralization (Zeta potential: EPS-Ca > Ca-EPS, Table 1). Enhanced Ca^2+^ mediated charge neutralization in EPS-Ca, promoting larger aggregate formation. At elevated Ca^2^^+^ concentrations, size ranges expanded and distributions shifted further right, demonstrating intensified aggregation. Furthermore, the filtration analysis of suspensions prepared by mixing the EPS and Ca^2+^ solutions revealed that nearly 100% of Ca^2+^ were complexed with the EPS, as determined by measuring free Ca^2+^ concentrations in the filtrate. This binding efficiency remained unaffected by the order of Ca^2+^ addition, confirming that the mixing sequence does not influence Ca^2+^ utilization efficiency within the studied concentration range.

**Figure 3:**
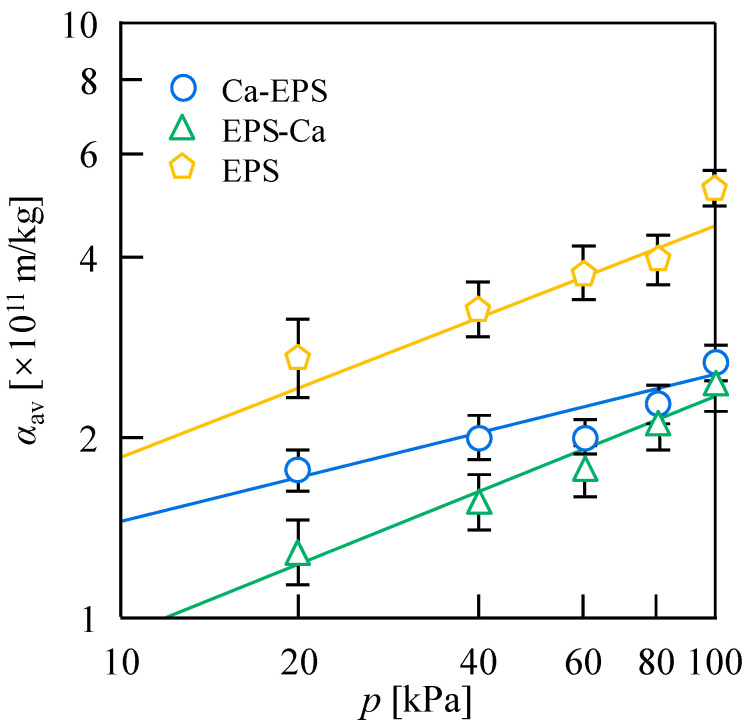
Relationship between average filtration resistance, *α*_av_, and filtration pressure, *p*, for the EPS, Ca-EPS, and EPS-Ca. Here, Ca-EPS or EPS-Ca were the suspended substances formed by the interaction between Ca^2+^ (1 mM) and EPS (1.0 g/L).

Figure 3 plots the logarithmic average filtration resistance, *α*_av_, values versus filtration pressure, *p*, where *α*_av_ was calculated from Ruth plots (Figure S1a–e) using Equation (3). The results demonstrate that the filtration resistance consistently follows the order EPS > Ca-EPS > EPS-Ca across all filtration pressures. EPS-Ca exhibits the lowest *α*_av_ value and optimal filterability under identical pressures, while the pure EPS shows the highest *α*_av_ and poorest filtration performance. Combined with Equation (4), EPS-Ca displays a faster pressure-dependent increase in filtration resistance compared to Ca-EPS, indicating greater compressibility (a higher *n* value) of the EPS-Ca filter cake. At elevated pressures (>80 kPa), *α*_av_ values of EPS-Ca and Ca-EPS converge as the filter cake reaches compression limits, with tightly packed particles minimizing resistance differences between addition orders. While high pressure does not alter polymer structure, it enhances particle redistribution and reduces the porosity of the filter cake, ultimately increasing filtration resistance [3]. These results indicate that excessive filtration pressure can over-compress the cake layer, thereby decreasing porosity while elevating filtration resistance.

**Figure 4:**
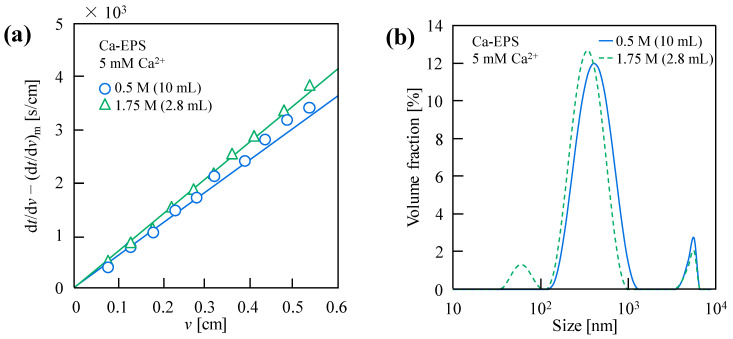
(**a**) UF behavior and (**b**) typical particle size distribution of Ca-EPS formed using both Ca^2+^ dosage modes [0.5 M (10 mL), 1.75 M (2.8 mL)]. Filtration was performed at 20 kPa with a 10 kDa UF membrane, and the final EPS and Ca^2^^+^ concentrations in the mixed solution were 1.0 g/L and 5 mM, respectively.

Figure 4a demonstrates the UF behavior of Ca-EPS under varying Ca^2+^ addition strategies. The high-volume, low-concentration Ca^2+^ addition mode exhibits lower filtration resistance, likely attributable to larger aggregate formation, as evidenced by the rightward peak shift in particle size distributions (Figure 4b). Zeta potential analysis (Table 1) reveals weaker intermolecular repulsion in these systems with the high-volume, low-concentration Ca^2+^ addition mode because of higher Zeta potential values [−5.99 mV for 0.5 M (10 mL) vs. −7.47 mV for 1.75 M (2.8 mL)], confirming enhanced Ca^2+^-mediated charge neutralization. This charge screening reduces electrostatic repulsion, promoting particle aggregation into larger structures that minimize dispersion, thereby lowering filtration resistance.

### 3.2. Effects of Ca^2+^ Mixing Sequence, Dosing Volume, and Flow Rate on SA Filtration Behavior

**Figure 5:**
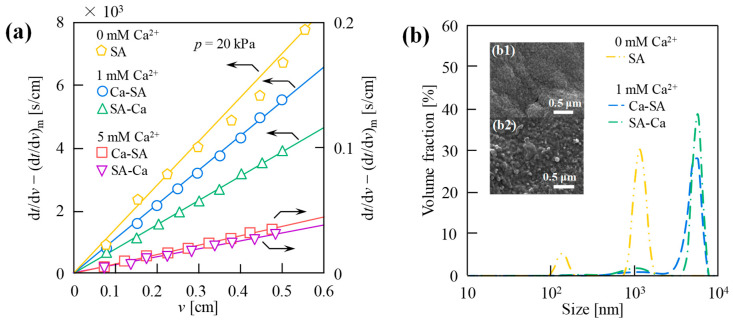
The effect of Ca^2^^+^ addition order (0, 1, and 5 mM) on the (**a**) UF behavior and (**b**) particle size distribution of sodium alginate (SA), Ca-SA, and SA-Ca. Here, the filtration of 1.0 g/L SA concentration was conducted at 20 kPa using a 10 kDa UF membrane. Ca-SA, the suspended substance formed by adding Ca^2+^ solution to SA solution (Ca^2+^ → SA). SA-Ca, the suspended substance formed by adding SA solution to Ca^2+^ solution (SA → Ca^2+^). The inset of Figure 5b shows the SEM images of (**b1**) Ca-SA and (**b2**) SA-Ca obtained for the case of 1 mM Ca^2^^+^. At 5 mM Ca^2+^, SA reacts completely with Ca^2^^+^, forming large flocs visible to the naked eye that fully precipitate; hence, their particle size distributions are not shown in the figure.

Investigations of filtration behavior in EPS and Ca^2+^ solutions with distinct addition protocols revealed distinct differences. This prompted systematic analysis employing the model EPS (SA) to evaluate the impact of addition sequence and metal ion coordination schemes. This study uncovered notable sequence-dependent interactions between SA and Ca^2+^, demonstrating that the ionic binding sequence fundamentally governs macromolecular assembly and filtration performance. Figure 5a compares UF behaviors of SA, SA-Ca, and Ca-SA under different mixing sequences. Consistent with EPS-Ca^2+^ interaction patterns, all Ca^2+^-modified systems exhibited reduced filtration resistance versus Ca^2+^-free SA, irrespective of sequence or concentration. SA-Ca demonstrated markedly lower filtration resistance than Ca-SA at both 1 mM and 5 mM Ca^2+^ levels. However, at 5 mM Ca^2+^, diminished differences in the filtration rate and resistance between sequences suggest the saturation of Ca^2+^ SA binding, neutralizing the operational impact of the addition order through complete complexation.

Figure 5b presents particle/colloid size distributions of SA, SA-Ca, and Ca-SA, alongside SEM images of Ca-SA (b1) and SA-Ca (b2). At 1 mM Ca^2+^, both Ca-SA and SA-Ca exhibited right-shifted particle size peaks relative to SA, indicating larger aggregate formation and reduced filtration resistance. SA-Ca consistently displayed right-shifted peaks compared to Ca-SA, suggesting the partial Ca^2+^ encapsulation of SA forming larger particles. As shown in the inset of Figure 5b, SEM analysis confirmed coarser aggregates in SA-Ca (Figure 5(b2)), absent in the more homogeneous Ca-SA (Figure 5(b1)). Notably, at 5 mM Ca^2+^, SA reacts completely with Ca^2+^, forming large flocs visible to the naked eye that fully precipitate, hence their particle size distributions are not shown in the figure. Consistent with previous studies [3,4,24], SA-Ca and Ca-SA at 5 mM Ca^2+^ exhibit minimal filtration resistance (Figure 5a).

**Figure 6:**
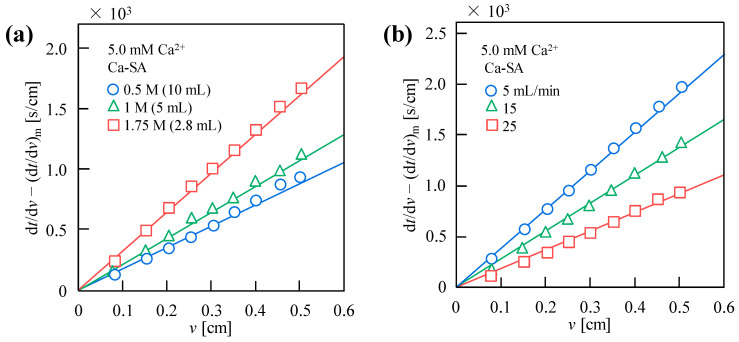
UF behavior of (**a**) 1.0 g/L SA solution treated with varying Ca^2^^+^ dosing methods [0.5 M (10 mL), 1 M (5 mL), 1.75 M (2.8 mL)] and (**b**) 10 mL 0.5 M Ca^2^^+^ added to 1000 mL SA solution at different flow rates (5, 15, 25 mL/min). All experiments were performed under 20 kPa using 10 kDa MWCO UF membrane, with final Ca^2^^+^ concentration standardized at 5 mM in all mixed solutions.

Figure 6a illustrates the UF behavior of Ca-SA with varying Ca^2^^+^ dosages. Filtration resistance decreased sequentially as 1.75 M (2.8 mL) > 1 M (5 mL) > 0.5 M (10 mL), demonstrating the critical influence of the dosing method on performance despite identical final concentrations. Low-concentration, high-volume Ca^2^^+^ addition notably minimized resistance, probably because of the increasing size of particles or colloid in suspension (Figure S2a). Minimal pH variations across mixing methods (Table 1) excluded significant pH-related effects. The Zeta potentials of −15.1 mV for 0.5 M (10 mL) were higher than that of −19.1 mV for 1.75 M (2.8 mL) (Table 1), likely due to intensified Ca^2^^+^-SA interactions promoting large gel network formation and narrower size distribution. This aggregation reduced suspended colloidal particles, concentrating the remaining free SA in solution and generating dual peaks in the 1.75 M sample (Figure S2a). In summary, within concentration–volume variation systems, the particle size in suspensions, Zeta potential, solution pH, and gel network formation may constitute the predominant factors influencing filtration resistance.

Figure 6b demonstrates the UF performance of Ca-SA under varying dosing flow rates, showing filtration resistance progression as 5 > 15 > 25 mL/min. With increasing Ca^2+^ dosing flow rates, size distributions (Figure S2b) reveal a rightward shift because of colloid coagulation caused by the decrease in the absolute value of zeta potentials (−29.0, −15.1 mV for 5, 25 mL/min, respectively). Elevated flow rates increased Ca^2^^+^-SA interactions, promoting calcium alginate formation through compressed electrical double layers [40]. This intensified flocculation decreased solution potential, enabling van der Waals-driven particle aggregation. The concurrent appearance of a 1000 nm peak at 25 mL/min suggests rapid Ca^2^^+^ influx induced poly-disperse aggregation states, with the minor peak likely corresponding to partial aggregates or nascent floc structures.

### 3.3. Mechanism Analysis of Mixing Modes Between EPSs and Ca^2+^

**Figure 7:**
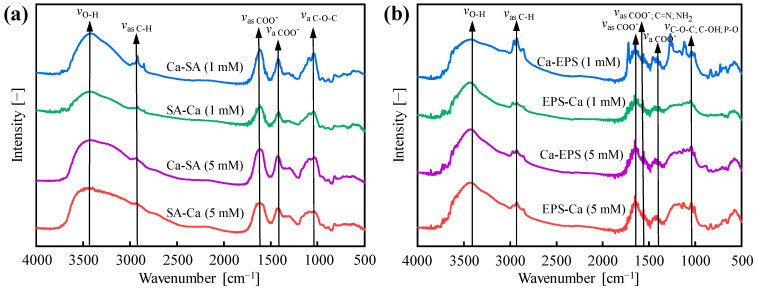
FTIR spectra of (**a**) Ca-SA/SA-Ca and (**b**) Ca-EPS/EPS-Ca prepared at Ca^2+^ concentrations of 1 and 5 mM.

Figure 7a,b present the FTIR spectra of Ca-SA/SA-Ca and Ca-EPS/EPS-Ca formed under varied mixing sequences and Ca^2+^ concentrations. Peak assignments demonstrate distinct molecular interactions between Ca^2^^+^ and biopolymers across mixing modes. Five characteristic peaks emerge in Ca-SA/SA-Ca (Figure 7a): 3400 cm^−1^ (*v*_O-H_), 2970 cm^−1^ (*v*_as C-H_), 1606 cm^−1^ (*v*_as COO_^−^), 1407 cm^−1^ (*v*_a COO_^−^) and 1055 cm^−1^ (*v*_a C-O-C_) [5,47]. Ca-EPS/EPS-Ca (Figure 7b) exhibits six peaks: 3400 cm^−1^ (*v*_O-H_), 2970 cm^−1^ (*v*_as C-H_), 1640 cm^−1^ (*v*_as COO_^−^), 1545 cm^−1^ (*v*_as COO_^−^_; C=N; NH_2__), 1400 cm^−1^ (*v*_a C=O_), and 1055 cm^−1^ (*v*_C-O-C; C-OH; P-O_) [5,53,54].

FTIR analysis revealed distinct Ca^2+^ concentration-dependent spectral responses. At lower Ca^2+^ levels, SA-Ca samples showed minimal shifts in carboxyl peaks (1407/1606 cm^−1^), suggesting Ca^2+^ partially encapsulating SA reduces free SA availability, thereby mitigating membrane fouling. Conversely, Ca-SA samples exhibited intensified C-H stretching at 2970 cm^−1^, demonstrating that tighter Ca^2+^ SA binding enhances fatty acid exposure. High Ca^2+^ concentrations diminished peak shift variations across addition sequences, indicating that dominant ion–polymer interactions override mixing order effects. For EPSs, EPS-Ca samples displayed smaller 1055/1400 cm^−1^ peak shifts versus Ca-EPS at low Ca^2+^ levels, attributed to Ca^2+^-induced EPS encapsulation forming filtration-favorable aggregates. The intensified 2970 cm^−1^ signal in Ca-EPS reflects enhanced fatty acid exposure through Ca^2+^-mediated crosslinking. Elevated Ca^2+^ concentrations minimized addition sequence impacts, demonstrating that high Ca^2+^ concentrations dominate EPS aggregation dynamics and filtration performance.

**Figure 8:**
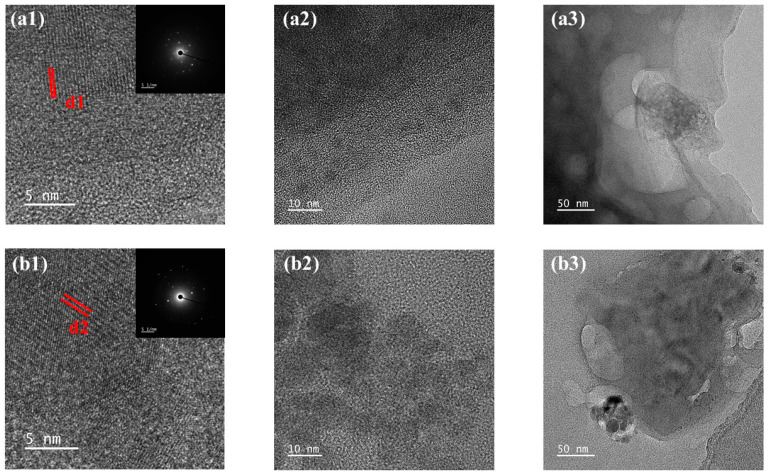
Transmission electron microscopy (TEM) images and selected area electron diffraction patterns of (**a1**–**a3**) Ca-SA and (**b1**–**b3**) SA-Ca prepared at a Ca^2+^ concentration of 1 mM, relative to the scales of 5, 10, and 50 nm, respectively.

Figure 8 presents TEM images and selected area electron diffraction (SAED) patterns comparing Ca-SA and SA-Ca. TEM micrographs of Ca-SA and SA-Ca reveal structural differences through particle spacing analysis and SAED pattern characteristics. SA-Ca exhibits larger Ca^2+^-induced spacing between polymeric components, evidenced by increased electron diffraction ring radii and expanded interpolymer distances compared to Ca-SA (Figure 8(a1,b1)). This structural evolution is further demonstrated in Figure 8(a2,a3,b2,b3) through comparative magnification analysis, showing SA-Ca’s tendency to form larger polymeric aggregates. These observations suggest Ca^2+^ partially coats SA molecules in SA-Ca, forming bulkier particles that reduce free SA concentration in solution relative to Ca-SA. Consequently, the SA-Ca configuration demonstrates reduced filtration resistance and mitigated membrane fouling potential.

**Figure 9:**
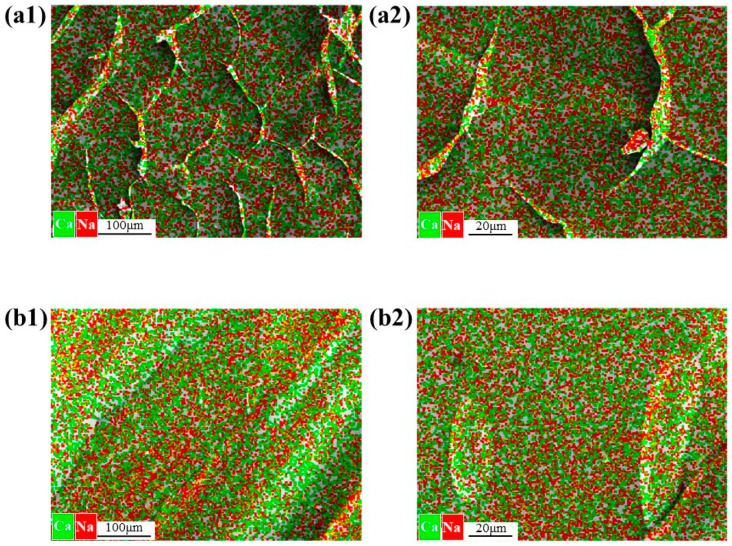
Scanning electron microscope (SEM) elemental analysis of (**a1**,**a2**) Ca-SA and (**b1**,**b2**) SA-Ca prepared at Ca^2+^ concentration of 1 mM, relative to scales of 100 and 20 μm, respectively.

Figure 9 presents the scanning electron microscopy (SEM) elemental analysis of Ca-SA and SA-Ca. The SA-Ca configuration exhibits densely concentrated Ca^2+^ distribution, particularly localized on particle surfaces, indicating Ca^2+^ SA binding that forms larger aggregates with tight crosslinking compared to Ca-SA. Comparative elemental mapping at 50 μm resolution (Figure 9(a2,b2)) reveals SA-Ca exhibits significantly higher Ca^2+^/Na^+^ ratios than its Ca-SA counterparts, confirming Ca^2+^’s dominant structural role in SA-Ca particle formation through preferential ionic interactions.

**Figure 10:**
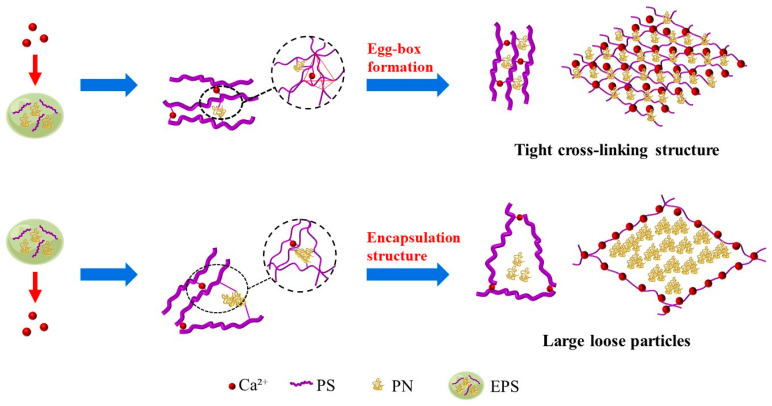
Structure formation mechanisms of EPS-Ca^2^^+^ interaction modes.

Figure 10 elucidates the structural formation mechanisms of EPS-Ca^2+^ interaction modes. EPS components—primarily polysaccharides (PSs) and proteins (PNs)—exhibit sequence-dependent behaviors under low Ca^2+^ concentrations. The EPS → Ca^2+^ addition sequence demonstrates reduced filtration resistance and accelerated filtration rates. Incomplete Ca^2+^ EPS interactions in EPS-Ca enable partial EPS coating by Ca^2+^, forming larger aggregates through coordination with anionic functional groups (e.g., carboxyls) in PS, as described by the “egg-box” gel model [55]. This incomplete binding diminishes gel network formation while lowering soluble EPS concentration, allowing larger particles or colloids to form on membranes with reduced cake resistance. Conversely, Ca-EPS obtained by the Ca^2+^ → EPS addition sequence achieves thorough Ca^2+^ EPS crosslinking, generating compact gel networks that retain finer EPS particulates in solution. These smaller particles deposit on membrane surfaces, amplifying filtration resistance and fouling severity. The findings confirm that ionic addition sequence critically governs EPS-Ca^2+^ interaction patterns and subsequent filtration performance.

Building on the sequence-dependent binding dynamics between EPS and Ca^2+^ that govern membrane fouling mitigation at low concentrations, systematic pressure-variable filtration tests were conducted. As operational pressure increased, EPS-Ca and Ca-EPS exhibited converging filtration resistances, with EPS-Ca demonstrating higher compressibility indices (*n* values) indicative of greater filter cake deformability. Elevated pressures amplified hydraulic compression effects, driving rapid resistance escalation through denser particulate packing at membrane interfaces. At critical pressure thresholds, interparticle forces and spatial reorganization override initial sequence-dependent structural differences, yielding comparable resistance profiles. While pressure leaves polymer architectures unaffected, it enhances particle redistribution and reduces the porosity of the filter cake, ultimately increasing filtration resistance regardless of the ionic addition sequence.

## 4. Conclusions

This study established that the Ca^2^^+^ concentration, mixing sequence with EPSs, and flow rate critically influence filtration resistance and membrane fouling under defined conditions. Sequence-dependent effects dominated at low Ca^2+^ concentrations: EPS → Ca^2+^ ordering reduced resistance through partial EPS encapsulation via incomplete Ca^2+^ binding, limiting free EPS availability for membrane fouling. Conversely, Ca^2+^ → EPS sequencing enabled full Ca^2+^ EPS crosslinking, generating dense networks while elevating residual free EPS levels that exacerbated membrane fouling. These sequence disparities diminished at elevated Ca^2+^ concentrations, where surplus ions saturated all EPS binding sites regardless of addition sequence. Pressure-dependent analyses revealed that EPS-Ca exhibited higher cake compressibility, where high pressure enhanced particle redistribution and reduced the porosity of the filter cake. The mixing sequence showed no significant influence on Ca^2+^ utilization efficiency within the studied concentration range. Low-concentration, high-volume Ca^2+^ addition effectively minimized the filtration resistance of SA. Elevated flow rates enhanced Ca^2+^ SA interactions, leading to the formation of large calcium alginate aggregates and consequently lower filtration resistance. The FTIR analysis of SA-Ca complexes revealed that the partial Ca^2+^ encapsulation of SA reduced free SA availability, thereby mitigating membrane fouling. Compared to Ca-EPS, EPS-Ca samples exhibited smaller peak shifts at 1055/1400 cm^−^^1^ under low Ca^2+^ conditions, suggesting Ca^2+^-induced EPS encapsulation and the formation of aggregates favorable for filtration. In SA-Ca systems, Ca^2+^ partially coated SA molecules, forming bulkier particles that decreased free SA concentration in solution relative to Ca-SA. SA-Ca demonstrated significantly higher Ca^2+^/Na^+^ ratios than Ca-SA, confirming Ca^2+^’s dominant structural role in particle formation through preferential ionic interactions. In EPS-Ca, incomplete Ca^2+^ EPS interactions allowed partial EPS coating by Ca^2+^, facilitating larger aggregate formation via coordination with anionic functional groups (e.g., carboxyls) in PS. These findings advance the mechanistic understanding of EPS-related membrane fouling in resource recovery processes while proposing novel mitigation strategies through the systematic control of ionic interaction variables. Future work will focus on (1) optimizing characterization methods, (2) elucidating EPS–metal ion interaction mechanisms, and (3) assessing versatility across membrane types.

## Figures and Tables

**Table 1:** Experimental parameters and characteristics of the sample preparation in this study. Here, *C*_0-Ca_ and *V*_0-Ca_ are the initial concentration and volume of Ca^2+^ solution, respectively; *C*_0-EPS_ and *V*_0-EPS_ are the initial concentration and volume of EPS solution, respectively; *Q* is the volumetric mixing flux; *C*_Ca_ is the final concentration of Ca^2^^+^ in the mixed suspension; a final EPS concentration, *C*_EPS_, of 1.0 g/L was used in the mixed solution for all experiments.

Mixing Order	Ca^2+^ Solution		EPS Solution		*Q* [mL/min]	*C*_Ca_ [mM]	pH	Zeta [mV]
	*C*_0-Ca_ [mM]	*V*_0-Ca_ [mL]	*C*_0-EPS_ [g·L^−1^]	*V*_0-EPS_ [mL]				
Ca^2+^ → EPS	5	50	1.25	200	25	1	6.66	−13.2
	25					5	7.10	−7.82
	500	10	1.0	1000			7.12	−5.99
	1750	2.8					7.19	−7.47
EPS → Ca^2+^	5	50	1.25	200		1	7.25	−11.5
	25					5	7.23	−8.62
Ca^2+^ → SA	500	10	1.0	1000			6.07	−15.1
	1000	5					6.18	−16.8
	1750	2.8					6.22	−19.1
	500	10			5		7.30	−29.0
					15		7.05	−18.7
	5	50	1.25	200	25	1	6.79	−19.0
	25					5	6.57	−7.66
SA → Ca^2+^	5					1	7.02	−6.3
	25					5	6.47	−6.84

## Data Availability

The original contributions presented in this study are included in the article. Further inquiries can be directed to the corresponding author.

## Supplementary Materials

The following supporting information can be downloaded at: https://www.mdpi.com/article/10.3390/membranes15060169/s1. Figure S1: Ultrafiltration behavior of EPS, Ca-EPS, and EPS-Ca at various pressures. Figure S2: (a) Size distributions of Ca-SA with different Ca^2+^ dosages [0.5 M (10 mL), 1.0 M (5 mL), and 1.75 M (2.8 mL)]; (b) Size distributions of Ca-SA with different dosing flow rates (5, 15, 25 mL/min). Table S1: Dead-end filtration properties in microfiltration (MF), ultrafiltration (UF), and nanofiltration (NF) of sodium alginate (SA) with Ca^2+^ addition.
