# Supplementary material for: Calcium Ion Mixing Modes Govern Membrane Fouling Mitigation During Membrane-Based Recovery of Extracellular Polymeric Substances
Source: Membranes (Basel). 2025 Jun 5;15(6):169. doi: 10.3390/membranes15060169 (PMC12194901; doi:10.3390/membranes15060169)
Supplement: Supplementary file 1 [file membranes-15-00169-s001.zip › membranes-3642304-supplementary.pdf]

Supplementary Material

Calcium ion mixing modes govern membrane fouling mitigation during membrane-based recovery of extracellular polymeric substances

Da-Qi Cao[a*], Yi-Xuan Song[a], Yun-Feng Wu[a], Guri Yihuo[a], Jing-Yi Jin[a]

[a]Sino-Dutch R&D Centre for Future Wastewater Treatment Technologies/Key Laboratory of Urban Stormwater System and Water Environment, Beijing University of Civil Engineering and Architecture, Beijing, 100044, China

[*]Corresponding authors: D.-Q. Cao (caodaqi18@163.com).

Table S1 Dead-end filtration properties in microfiltration (MF), ultrafiltration (UF), and nanofiltration (NF) of sodium alginate (SA) with Ca^{2+} addition. Here, MW, molecular weight; K_v, Ruth filtration coefficient; α_{av}, cake average specific resistance.

Membrane type	Pressure	pH	MW of SA [kDa]	Con. of SA [g/L]	Con. of Ca^{2+} [mM]	K_v [cm^2/s]	α_{av} [m/kg]	Ref.
NF	350	7	12–80	0.03	0	2.24×10^{-2}	1.166×10^{16}	Listiarini et al., 2009
					1	3.52×10^{-2}	7.417×10^{15}	
					3	5.01×10^{-2}	5.211×10^{15}	
					6	6.34×10^{-2}	4.118×10^{15}	
MF (1, 4, 7 μm)	20	6.9–7.1	120–190	2.0	2	7.80×10^{-5}	8.604×10^{10}	Cao et al., 2018b
					4	7.88×10^{-4}	8.517×10^{9}	
					6	7.69×10^{-3}	8.727×10^{8}	
UF (10 kDa)	10–100	6.9–7.1	120–190	1.0	1	6.20×10^{-5}	2.165×10^{8}	Cao et al., 2017
					2	1.34×10^{-4}	1.002×10^{8}	
					4	8.00×10^{-4}	1.678×10^{7}	
					6	3.92×10^{-3}	3.424×10^{6}	
					8	1.77×10^{-2}	7.584×10^{5}	
					10	4.17×10^{-2}	3.219×10^{5}	
UF (140 kDa) MF (0.1 μm)	40	7.0	–*	1.0	0.5	–	0.8×10^{14}	Zhang et al., 2017
					1		1.45×10^{15}	
					1.5		3.5×10^{15}	
					2		1.55×10^{15}	
					2.5		2.5×10^{14}	

					3		1.5×10^{14}	
UF (140 kDa) MF (0.2 μm)	40	7.0	32–250	0.75	1	4.43×10^{-4}	2.398×10^{15}	Zhang et al., 2018
UF (10 kDa)	20	7.0	120–190	1.0	0	3.33×10^{-5}	4.031×10^{8}	Cao et al., 2020
		6.8			1	6.25×10^{-5}	2.148×10^{8}	
		6.4			2	1.33×10^{-4}	1.009×10^{8}	
UF (0.1 μm)	130	6.4	–	0.05	0	4.85×10^{-1}	1.20×10^{14}	Lin et al., 2022
		6.0			0.02	3.06×10^{-1}	1.90×10^{14}	
		5.8			0.05	1.11×10^{-2}	5.24×10^{15}	
		5.7			0.5	1.80×10^{-2}	3.24×10^{15}	
		5.6			2.0	3.13×10^{-2}	1.86×10^{15}	
		5.75			3.0	5.82×10^{-2}	1.00×10^{15}	
		5.8			6.0	3.06×10^{-1}	1.90×10^{14}	

*No data provided in the literature.

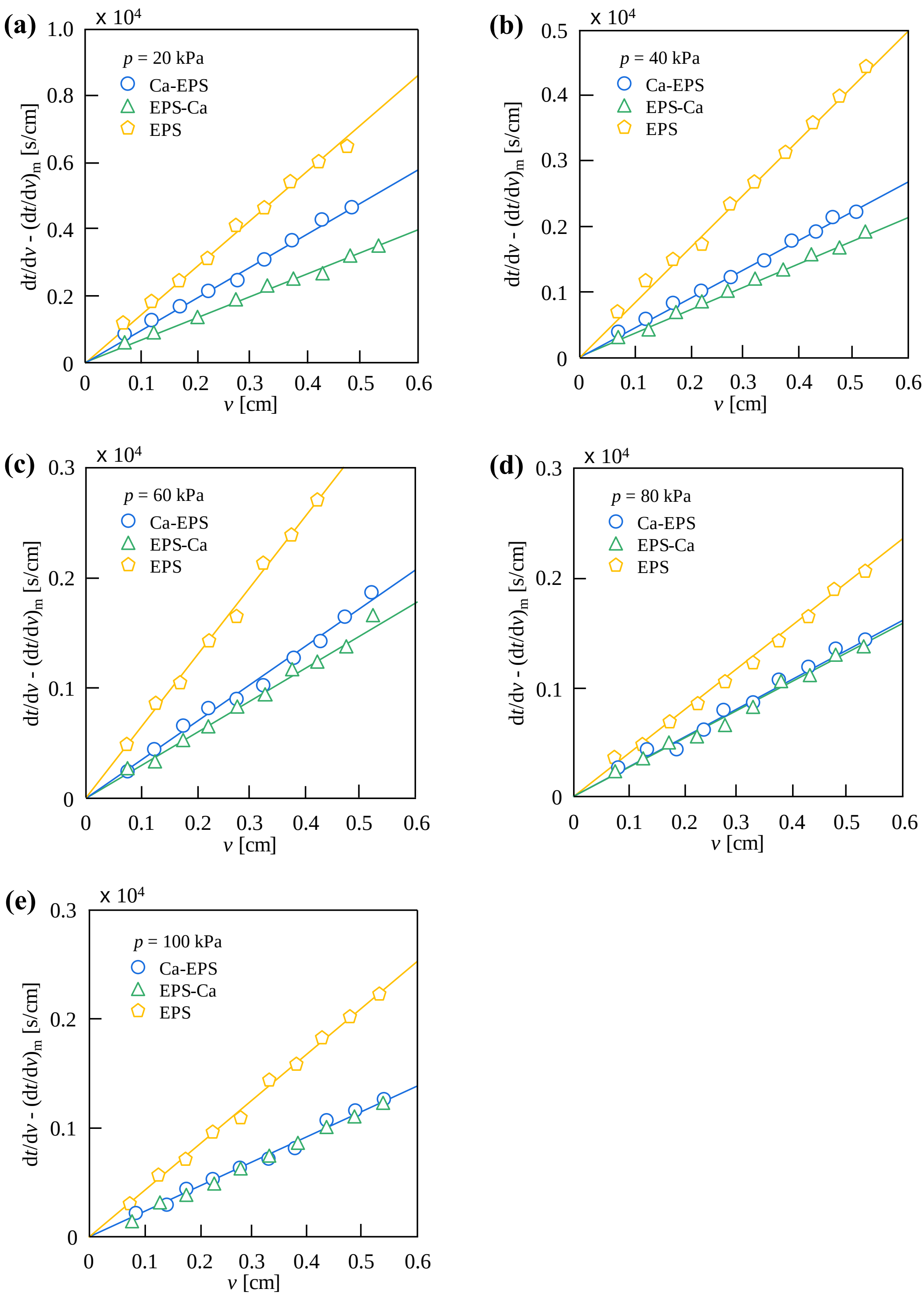

Figure S1 Ultrafiltration behavior of EPS, Ca-EPS, and EPS-Ca at various pressures: (a) 20 kPa; (b) 40 kPa; (c) 60 kPa; (d) 80 kPa; (e) 100 kPa. Here, Ca-EPS or EPS-Ca were the suspended substances formed by the interaction between Ca^{2+} (1 mM) and EPS (1.0 g/L).

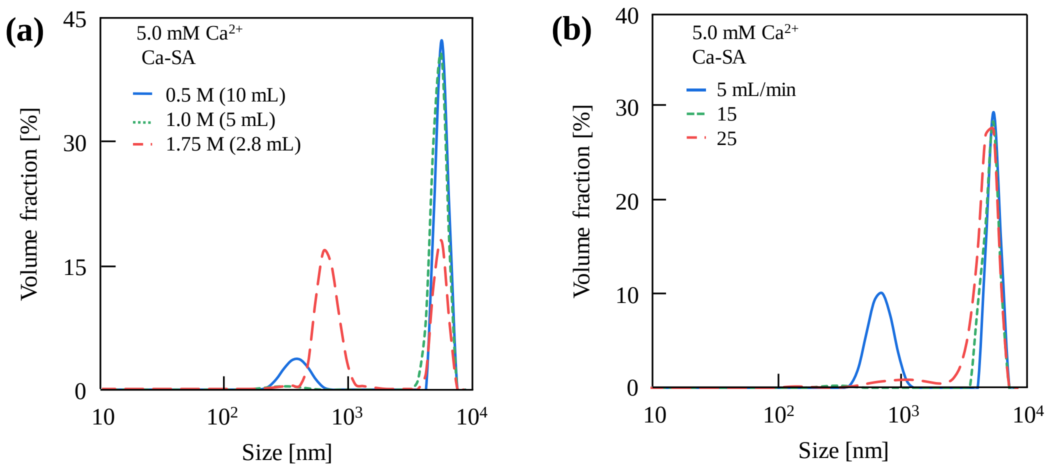

Figure S2 (a) Size distributions of Ca-SA with different Ca^{2+} dosages [0.5 M (10 mL), 1.0 M (5 mL), and 1.75 M (2.8 mL)]; (b) Size distributions of Ca-SA with different dosing flow rates (5, 15, 25 mL/min). Filtration was conducted at 20 kPa using a10 kDa UF membrane, and the final Ca^{2+} concentration in the mixed solution was 5 mM.

References

D.-Q. Cao, X.-D. Hao, Z. Wang, X. Song, E. Iritani, N. Katagiri, Membrane recovery of alginate in an aqueous solution by the addition of calcium ions: Analyses of resistance reduction and fouling mechanism, J. Membr. Sci. 535 (2017) 312−321, https://doi.org/10.1016/j.memsci.2017.04.050.

D.-Q. Cao, X. Song, X.-D. Hao, W.-Y. Yang, E. Iritani, N. Katagiri, Ca^{2+}-aided separation of polysaccharides and proteins by microfiltration: Implications for sludge processing, Sep. Purif. Technol. 202 (2018) 318-325, https://doi.org/10.1016/j.seppur.2018.03.070Get rights and content.

D.-Q. Cao, J.-Y. Jin, Q.-H. Wang, X. Song, X.-D. Hao, E. Iritani, N. Katagiri, Ultrafiltration recovery of alginate: Membrane fouling mitigation by multivalent metal ions and properties of recycled materials, Chinese J. Chem. Eng. 28 (2020) 2881−2889, https://doi.org/10.1016/j.cjche.2020.05.014.

K. Listiarini, W. Chun, D.-D. Sun, J.-O. Leckie, Fouling mechanism and resistance analyses of systems containing sodium alginate, calcium, alum and their combination in dead-end fouling of nanofiltration membranes, J. Membr. Sci. 344 (2009) 244–251, https://doi.org/10.1016/j.memsci.2009.08.010Get rights and content.

L. Lin, M. Zheng, C. Ma, Q. Fu, Y. Zhang, Transparent exopolymer particles-associated membrane fouling analyses of systems containing sodium alginate, calcium, iron, alum and their combination during dead-end ultrafiltration, J. Clean Prod. 366 (2022) 132983, https://doi.org/10.1016/j.jclepro.2022.132983.

M.-J. Zhang, H.-C. Hong, H.-J. Lin, L.-G. Shen, H.-Y. Yu, G.-C. Ma, J.-R. Chen, B.-Q. Liao, Mechanistic insights into alginate fouling caused by calcium ions based on terahertz time-domain spectra analyses and DFT calculations, Water Res. 129 (2018) 337–346, https://doi.org/10.1016/j.watres.2017.11.034.

M.-J. Zhang, H.-J. Lin, L.-G. Shen, B.-Q. Liao, X.-L. Wu, R.-J. Li, Effect of calcium ions on fouling properties of alginate solution and its mechanisms, J. Membr. Sci. 525 (2017) 320–329, https://doi.org/10.1016/j.memsci.2016.12.006.
